# Intraventricular tumours: An individual patient data meta-analysis of conventional craniotomy *versus* minimally invasive techniques

**DOI:** 10.1016/j.bas.2026.107467

**Published:** 2026-10-06

**Authors:** Asfand Baig Mirza, Ariadni Georgiannakis, Sami Rashed, Joe Priestley, Feras Fayez, Pak Yin Lam, Amisha Vastani, Natalie Simon, Soha Zahid, Yasir A. Chowdhury, Prajwal Ghimire, Keyoumars Ashkan, Francesco Vergani, Ranjeev Bhangoo, Bassel Zebian, Sanjeev Bassi, Jonathan Pollock, Cristina Bleil, Jose-Pedro Lavrador

**Affiliations:** a Department of Neurosurgery, Queen's Hospital Romford, Barking, Havering and Redbridge University Hospitals NHS Trust, England, Essex, United Kingdom; b Barts and the London School of Medicine, Queen Mary University of London, England, London, United Kingdom; c School of Medicine and Population Health, The University of Sheffield, England, Sheffield, United Kingdom; d Imperial College Healthcare NHS Trust, England, London, United Kingdom; e King's College London GKT School of Medical Education, England, London, United Kingdom; f Department of Neurosurgery, King's College Hospital NHS Foundation Trust, England, London, United Kingdom

**Keywords:** Intraventricular tumours, Ependymoma, Neurocytoma, Surgical outcomes

## Abstract

**Introduction:**

Intraventricular Tumours (IVTs) are rare intracranial lesions primarily treated with surgery. Surgical modalities include conventional craniotomy and minimally invasive surgery (MIS), such as tubular-assisted and endoscopic surgeries, with unclear benefits associated to each approach.

**Research question:**

This systematic review compares gross total resection (GTR) rates, predictors of GTR, post-operative complications and requirement for permanent cerebrospinal fluid (CSF) diversion in conventional and MIS surgery.

**Material and methods:**

Five databases were searched until November 2024, following the PRISMA guidelines. Studies reporting individual-level outcome data for IVT surgery were included.

**Results:**

In total, 361 studies (1500 patients) were included. Most patients underwent conventional craniotomy (n = 1,243, 83%), followed by endoscopic (n = 223, 15%) and tubular-assisted surgery (n = 34, 2.3%). GTR was achieved in 67% (n = 830) of conventional cases compared to 46% (n = 103) of endoscopic and 76% (n = 26) of tubular-assisted approaches (p **<** 0.001 in univariate analysis; OR 0.38, 95% CrI 0.13–1.13 in multivariable analysis). Complications were reported in 37.8% (n = 567) of patients, among whom 48.9% (n = 277) experienced at least one complication, with most undergoing conventional craniotomy (n = 239). Permanent CSF diversion was required in 65% (n = 277) of patients and was significantly more likely in patients undergoing MIS in univariate analysis. The multivariable analysis revealed that only pre-operative hydrocephalus (OR 8.92, 95% CrI 2.04-38.93) was significantly associated with permanent diversion, while surgical approach was not.

**Discussion and conclusion:**

There is a significant unbalance in the amount of cases reported with conventional craniotomy and MIS supporting the early days of MIS approaches for IVTs. Conventional and MIS approaches for IVTs achieve comparable rates of GTR and permanent CSF diversion.

## Highlights

• Minimally invasive surgery, has comparable resection rates, for IVTs , when compared with conventional craniotomy.
• Permanent post-operative CSF diversion is independent of surgical approach.
• Complications for IVTs are seen in both MIS and conventional craniotomy.

## Abbreviations:

CSF –: Cerebrospinal fluid
EoR –: Extent of resection
IVT –: Intraventricular tumour
PRISMA –: Preferred Reporting Items for Systematic Reviews and Meta-Analyses
PROSPERO –: International prospective register of systematic reviews
MIS –: Minimally invasive surgery
IPD –: Individual Patient Data
GTR –: Gross Total Resection

## 1 Introduction

Intraventricular tumours (IVTs) are rare intracranial lesions that account for 1.2% of primary brain tumours (Kirollos et al., 2019). Several histological types are recognized, and their distribution depends on age, with choroid plexus tumours, ependymomas and subependymomal giant cell astrocytomas being more common in children (Katz et al., 2012; Louis et al., 2016) and colloid cysts, subependymomas, central neurocytomas and meningiomas being more prevalent in adults (Waldron and Tihan, 2003). Despite most IVTs being benign, their location within the confined ventricular system can lead to significant clinical manifestations such as hydrocephalus. (Agarwal and Kanekar, 2016)^,^ (Jumah et al., 2020) A review of the most common tumour types encountered based on anatomic location can be seen in Table 1.

**Table 1:** Tumour group by anatomical location.

	Lateral Ventricle	Third Ventricle	Fourth Ventricle	Total
Glioma	196 (32%)	50 (14%)	80 (49%)	326 (28%)
Choroid Plexus Tumours	58 (9.3%)	23 (6.4%)	19 (12%)	100 (8.7%)
Neuroepithelial tumours	168 (27%)	14 (3.9%)	1 (0.6%)	183 (16%)
Meningioma	154 (25%)	15 (4.1%)	19 (12%)	188 (16%)
Embryonal tumours	2 (0.3%)	40 (11%)	27 (16%)	69 (6.0%)
Cysts and benign lesions	6 (1.0%)	174 (48%)	0 (0%)	180 (16%)
Other/miscellaneous	37 (6.0%)	46 (13%)	18 (11%)	101 (8.8%)
Total	621 (100%)	362 (100%)	164 (100%)	1147 (100%)

Surgical intervention is the cornerstone of management for IVTs that require treatment (Krishnatry, 2019; Chong et al., 2022). The primary goals of surgery include obtaining a definitive tissue diagnosis, re-establishing the cerebrospinal fluid (CSF) circulation and achieving therapeutic tumour resection, where appropriate (Vigo et al., 2021). Over time, various surgical approaches and trajectories to access the ventricular system have been developed and refined, including traditional conventional craniotomy, minimally invasive tubular-assisted surgery, and neuro-endoscopic techniques (Fig. 1). (Agarwal and Kanekar, 2016)

**Fig. 1:**
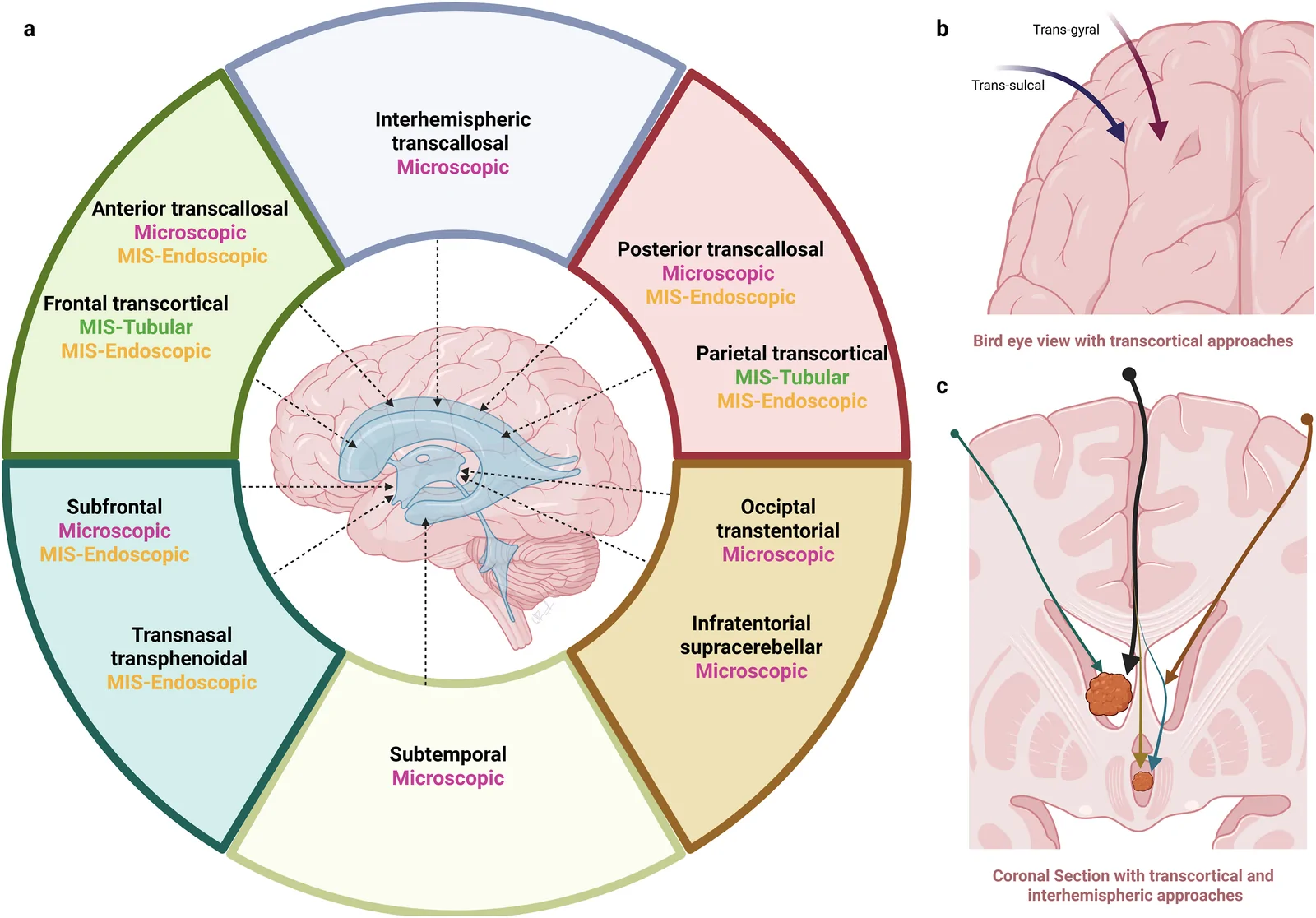
Summary of approaches for intraventricular tumours (A) 360-view of approaches to intraventricular tumours with corresponding nomenclatures of approaches and techniques associated with them. Color coded techniques are highlighted with pink for microscopic, green for MIS tubular and orange for MIS endoscopic (B) Bird eye view highlighting transcortical approaches namely trans-gyral and trans-sulcal (C) Coronal section demonstrating transcortical and interhemispheric approaches to an intraventricular tumour (Illustrations created using Biorender.com).

Each surgical modality offers distinct advantages and disadvantages. Conventional craniotomy provides wide visualization and a multitude of surgical corridors (transcortical, transsulcal, interhemispheric and subfrontal) (Fig. 2). (Yaşargil and Abdulrauf, 2008) However, it has historically been associated with a risk of postoperative complications, particularly seizures (Al-Dorzi et al., 2017; Desai et al., 2002). Tubular-assisted surgery is less invasive and uses a narrow corridor to access deep-seated lesions, thereby reducing parenchymal disruption, intra-operative blood loss and recovery times (Eliyas et al., 2016). Endoscopic surgery offers the added benefit of continuous irrigation for improved haemostasis and debris clearance (Souweidane, 2005), better visualization and illumination in the depth of the ventricles (Schroeder, 2013), and the ability to perform concurrent procedures such as a third ventriculostomy to address hydrocephalus (Barber et al., 2013).

**Fig. 2:**
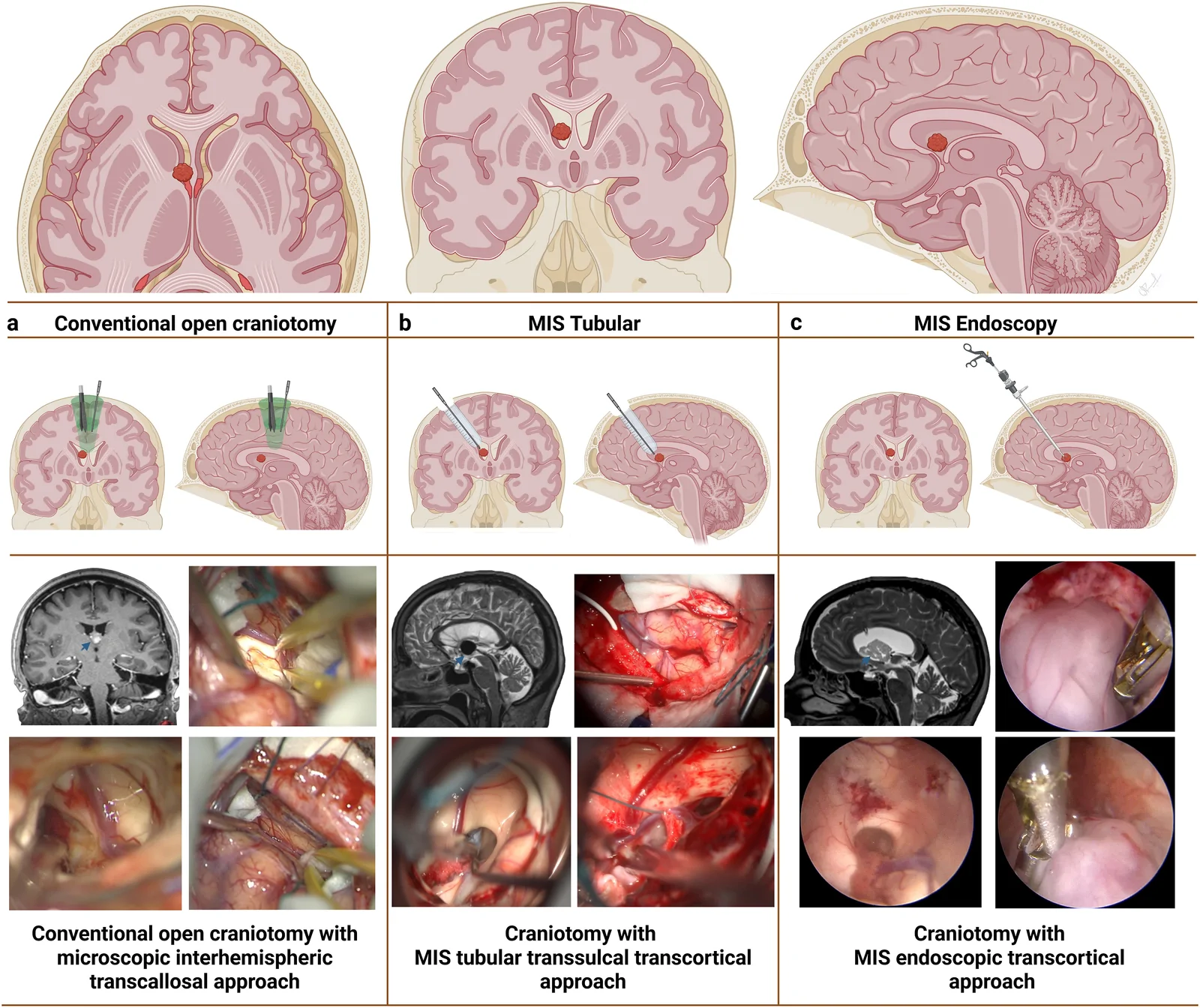
Comparison of conventional open craniotomy, MIS tubular and MIS endoscopic technique for intraventricular tumours (A) Illustrative and operative images for conventional open craniotomy followed by microscopic interhemispheric transcallosal approach for a third ventricular tumour (B) Illustrative and operative images for craniotomy followed by MIS tubular transsulcal transcortical approach for a third ventricular tumour (C) Illustrative and operative images for craniotomy followed by MIS endoscopic transcortical approach for a lateral ventricular cystic tumour (Illustrations created using Biorender.com).

The existing literature on the surgical management of IVTs is largely limited to retrospective case series, single-centre experiences, and case reports with no clear comparative analyses between different approaches, especially at the individual patient data (IPD) level. This systematic review and meta-analysis compares the odds of gross total resection (GTR), complication rates, and the need for permanent CSF diversion between conventional and minimally invasive surgery (MIS) approaches for IVTs.

## 2 Materials and methods

### 2.1 Study design and data collection

This systematic review and meta-analysis was registered on PROSPERO (CRD42024609691) and was conducted in accordance with the Preferred Reporting Items for Systematic Reviews and Meta-Analyses (PRISMA) guidelines (Page et al., 2021). MEDLINE, Web of Science, PubMed, Cochrane and Scopus were searched from inception to November 2024 for articles containing the terms: “ventricular”, “intraventricular” and “tumour”. Identified publications were added to Rayyan (Ouzzani et al., 2016). After deduplication, titles and abstracts were screened for eligibility by ABM, AG, PYL, and AV independently. Discrepancies were resolved by discussion with the senior authors. The reference lists of included studies were also screened. Studies were included if they reported individual-level surgical and outcome data on patients with intraventricular and choroid plexus tumours, undergoing surgery. Review articles, research proceedings and studies with no full text available were excluded.

Risk of bias was independently assessed using the ROBINS-I tool (Sterne et al., 2016) by PYL, JP, NS and SZ, and any disagreements were resolved by ABM.

A one-stage IPD meta-analysis was conducted to evaluate the association between surgical approach and GTR. Conventional surgery was defined as microsurgery performed via craniotomy, and MIS was defined as surgery performed with either endoscopic or tubular retractor-assisted systems (Aftahy et al., 2020; Sayehmiri et al., 2022). GTR was defined as 100% resection (Grutza et al., 2024). The secondary outcomes studied were predictors of GTR, complications and the need for permanent CSF diversion. Complications were categorised as intra-operative, immediate post-operative (≤48 h), and delayed post-operative, and were analysed both individually and as a composite “any complication” outcome. Permanent CSF diversion was defined as the need for a long-term diversion procedure (ventriculoperitoneal shunt, endoscopic third ventriculostomy with or without shunt, fenestration, or Ommaya reservoir). Variables for the multivariable model were selected *a priori* based on prior literature (Barber et al., 2013; Aftahy et al., 2020). Data extraction was performed in Microsoft Excel 365 (Microsoft Corporation, Redmond, USA) by AG, PYL, JP, NS and SZ and independently reviewed by ABM for quality and consistency.

### 2.2 Statistical analysis

A one-stage individual participant data (IPD) meta-analysis was conducted using Bayesian hierarchical logistic regression. The primary surgical approach model included study-level random intercepts and random treatment slopes to account for between-study variation; sensitivity and subgroup models used random intercepts only. Between-study heterogeneity was summarised by the posterior distribution of the random-effects standard deviation (τ), reported as the median and 95% credible interval (CrI). Model adequacy was assessed using posterior predictive checks.

Models were fitted using Hamiltonian Monte Carlo sampling via Stan (cmdstanr backend; 4 chains, 4000 iterations, 2000 warmup). Weakly informative priors were specified for all parameters: Normal(0,1) for fixed effects, Normal(0,1.5) for the intercept, Exponential(1) for between-study standard deviations, and LKJ(2) for the intercept-slope correlation. Convergence was evaluated using the potential scale reduction factor (R-hat < 1.01) and bulk effective sample size (ESS > 400); divergent transitions were addressed by refitting with increased target acceptance probability.

Results are reported as adjusted odds ratios (OR) with 95% CrI. Evidence for the direction of effects was quantified using the probability of direction (Pd), defined as the posterior probability that the effect is in the stated direction. Clinical relevance for the GTR predictor analysis was assessed using a pre-specified region of practical equivalence (ROPE) of OR 0.90–1.11, corresponding to <10% change in odds. This threshold was defined *a priori* on clinical grounds as the minimum difference considered meaningful in a high-risk context such as surgical decision-making (Kruschke, 2018). For the multivariable GTR predictor model, continuous predictors were standardised to a mean of 0 and SD of 1.

### 2.3 Missing data handling, model diagnostics and sensitivity analyses

Missing data were handled using multiple imputation by chained equations (mice; 10 datasets). Predictive mean matching, logistic regression, and polytomous regression were used for continuous, binary, and categorical variables, respectively. Bayesian estimates were pooled via posterior draw stacking; Rubin's rules were applied for frequentist analyses. Observed and imputed distributions were comparable across key variables.

Sensitivity analyses included restriction to complete cases, which produced qualitatively similar results to the multiply imputed analyses and are reported in the Supplementary Material S1. To assess robustness of the surgical approach estimate to confounding by indication, a propensity score analysis was conducted within each imputed dataset. The surgical approach was modelled using logistic regression with all available pre-specified covariates; the outcome model was refit using the logit-transformed propensity score in place of individual confounders. Propensity score overlap was assessed visually (Supplementary Material S2). The E-value was calculated for the surgical approach estimate to quantify the minimum unmeasured confounding required to explain the observed association. All analyses were performed in R (version 4.4.1; R Foundation for Statistical Computing, Vienna, Austria).

## 3 Results

### 3.1 Data quality and missingness

Multiple imputation (m = 10) was performed prior to all regression analyses. Variables with extensive missingness (KPS, length of stay, PFS, follow-up duration) were excluded from regression models. Observed and imputed distributions were compared using standard distributional tests; distributions were broadly consistent for most variables and all associations significant in the observed data remained directionally consistent following imputation. Tumour grade (observed p = 0.16; imputed p = 0.036) and pre-operative hydrocephalus (observed p = 0.057; imputed p = 0.005) showed distributional differences between datasets, consistent with recovery of underpowered associations given their respective missingness rates. Sex remained non-significant in both datasets (p = 0.30 and p = 0.16 respectively). Tumour size demonstrated a notable distributional shift in the endoscopic group following imputation (observed median 16 mm vs imputed median 41 mm), suggesting data were not missing at random in this subgroup; a sensitivity analysis excluding tumour size confirmed the primary surgical approach estimate was not materially influenced by this distribution (Supplementary Material S3). All subsequent regression analyses used imputed datasets.

### 3.2 Patient and study characteristics

The systematic search identified 36,780 articles of which 361 met inclusion criteria and contributed data for 1500 patients (Fig. 3). The median number of patients per study was 1 (IQR 1–3; range 1–59) and most studies were retrospective (351/361, 97%). The geographical and temporal distribution of included studies is presented in Fig. 4. Most studies had a moderate risk of bias (n = 271, 75.1%), followed by a low (n = 62, 17.1%) and a high risk (n = 28, 7.8%).

**Fig. 3:**
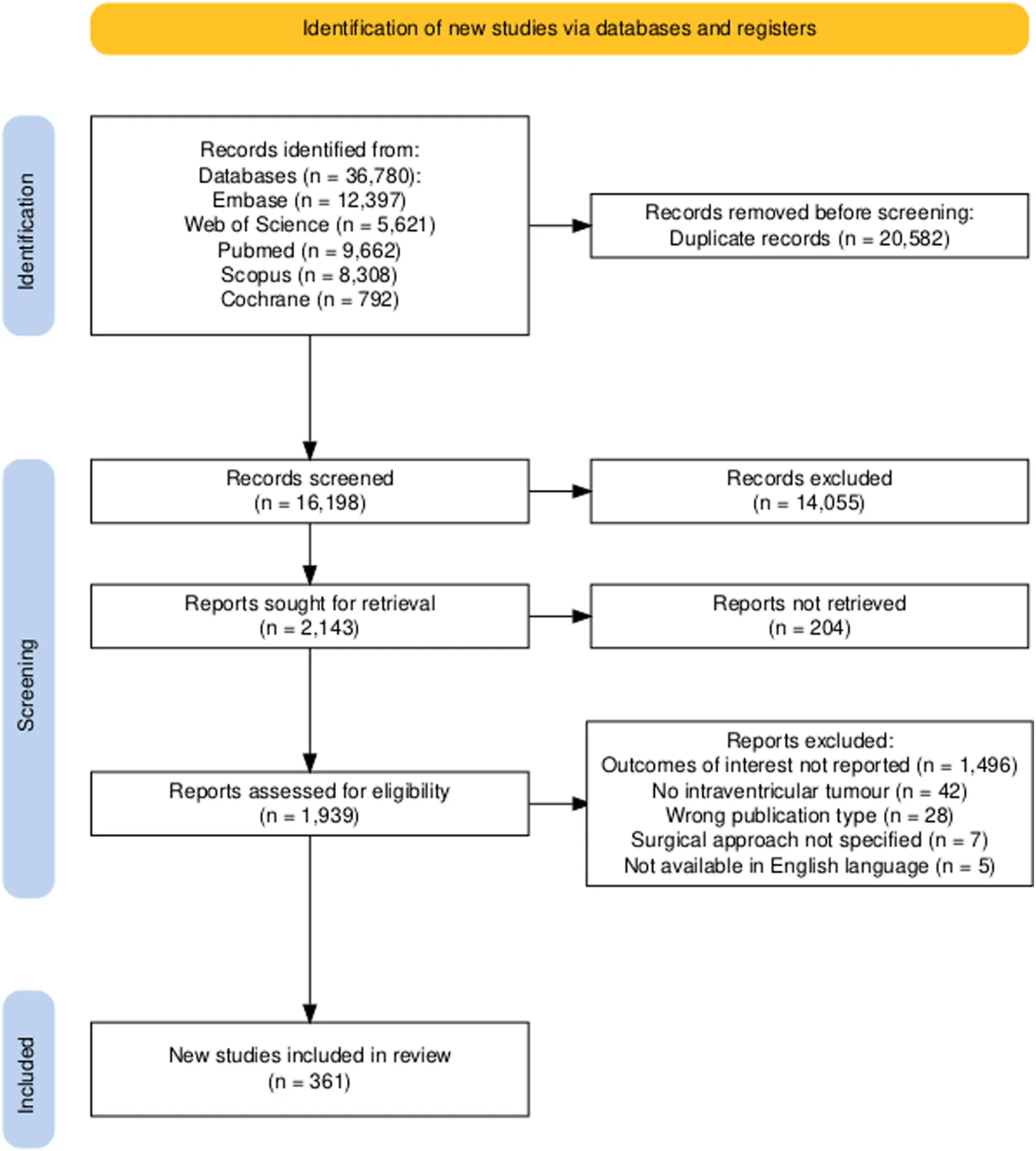
PRISMA flowchart of included studies.

**Fig. 4:**
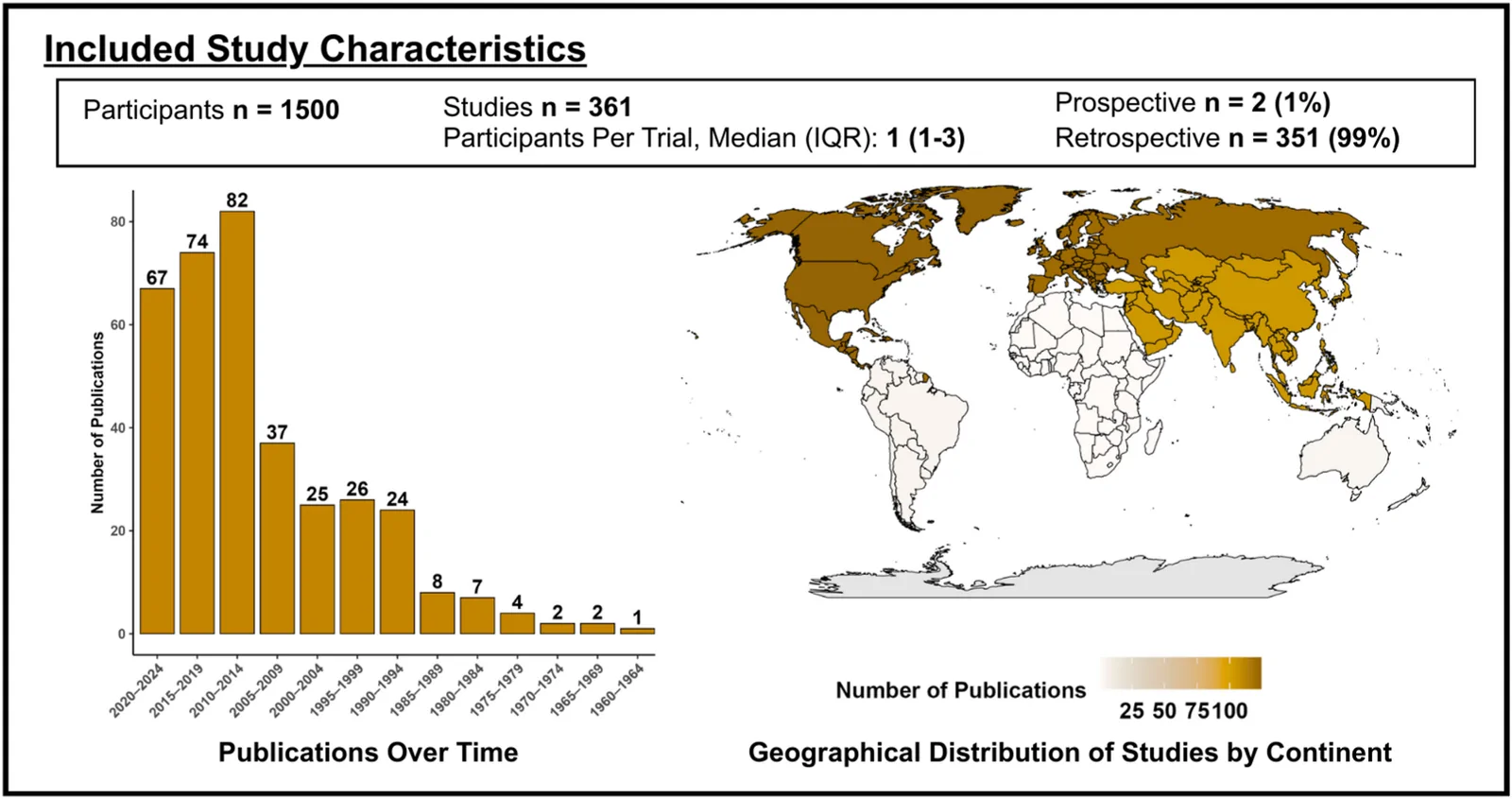
Characteristics of included studies.

### 3.3 Baseline patient characteristics by extent of resection (observed data)

Of 1500 participants, resection was performed in 1435 (95.7%) and biopsy ± CSF diversion in 65 (4.3%). Among resection cases, GTR was achieved in 959 (66.8%). Patients achieving GTR were older (28 years [IQR 13–47]) than those with non-GTR (22 years [IQR 8–39]; p < 0.001). Baseline functional status was similar between groups (KPS 90 [70–100] versus 90 [80–100]; p = 0.11), as was sex distribution (male 50% versus 52%; p = 0.30). Tumours in the GTR cohort were smaller (29 mm [15–50]) than non-GTR tumours (35 mm [20–56]; p = 0.015). Cystic tumours were more frequent among non-GTR patients (89% versus 75%; p = 0.003). Permanent CSF diversion was more frequent in non-GTR than GTR cases (76% versus 52%; p < 0.001).

Conventional craniotomy was the predominant approach (n = 1243; 83%), with endoscopic MIS in 223 cases (15%) and tubular retractor in 34 (2.3%). The distribution of extent of resection (EoR) differed significantly across approaches (p < 0.001). GTR rates were 67% (n = 830) for conventional craniotomy, 46% (n = 103) for endoscopic MIS, and 76% (n = 26) for tubular retractor; the EoR distribution differed significantly from conventional craniotomy for endoscopic cases (p < 0.001) but not tubular cases (p = 0.38). Most biopsies (91%) were performed endoscopically.

Baseline patient characteristics according to surgical approach are presented in Table 2. Imputed baseline data are provided in Supplementary Material S3.

**Table 2:** Baseline characteristics stratified by surgical approach (observed data).

Characteristic	N	Overall N = 1500	Conventional N = 1243	Endoscopic N = 223	Tubular N = 34	p (Conv vs MIS)	p (Conv vs Endoscopic)	p (Conv vs Tubular)
Extent of resection: Biopsy	1500	65 (4.3%)	6 (0.5%)	59 (26.5%)	0 (0.0%)	**<0.001**	**<0.001**	0.382
Extent of resection: GTR	1500	959 (63.9%)	830 (66.8%)	103 (46.2%)	26 (76.5%)	**<0.001**	**<0.001**	0.382
Extent of resection: Non-GTR	1500	476 (31.7%)	407 (32.7%)	61 (27.4%)	8 (23.5%)	**<0.001**	**<0.001**	0.382
Age	1456	25 (12, 44)	22 (10, 43)	30 (17, 44)	48 (32, 60)	**<0.001**	**<0.001**	**<0.001**
Baseline KPS	174	90 (70, 100)	90 (70, 100)	NA (NA, NA)	80 (80, 90)	0.794	-	0.794
Tumour size (mm)	476	30 (16, 50)	31 (20, 50)	16 (13, 56)	12 (12, 15)	**<0.001**	**0.023**	**<0.001**
Length of stay	116	6 (4, 10)	6 (5, 10)	8 (5, 8)	4 (2, 5)	**0.008**	0.497	**<0.001**
PFS	51	2 (1, 7)	2 (1, 6)	5 (2, 7)	12 (12, 12)	0.356	1.000	-
Follow-up (years)	805	2 (1, 4)	2 (1, 4)	1 (1, 2)	0 (0, 0)	**<0.001**	**<0.001**	**<0.001**
Tumour grade: High Grade	274	53 (19.3%)	50 (20.7%)	3 (10.3%)	0 (0.0%)	0.157	0.224	1.000
Tumour grade: Low Grade	274	221 (80.7%)	192 (79.3%)	26 (89.7%)	3 (100.0%)	0.157	0.224	1.000
Permanent CSF diversion	424	277 (65.3%)	186 (57.2%)	88 (91.7%)	3 (100.0%)	**<0.001**	**<0.001**	0.265
Male	1500	723 (48.2%)	589 (47.4%)	117 (52.5%)	17 (50.0%)	0.171	0.167	0.862
Cystic component	375	298 (79.5%)	187 (72.8%)	95 (94.1%)	16 (94.1%)	**<0.001**	**<0.001**	0.082
Pre-op hydrocephalus	1281	461 (36.0%)	366 (34.8%)	78 (39.4%)	17 (56.7%)	0.057	0.225	**0.019**
Raised ICP symptoms	1500	728 (48.5%)	598 (48.1%)	128 (57.4%)	2 (5.9%)	0.493	**0.011**	**<0.001**

Median (Q1, Q3) for continuous variables; n (%) for categorical variables. Percentages represent proportion within each surgical approach. Fisher's exact test for categorical variables; Wilcoxon rank-sum for continuous variables.

### 3.4 Primary outcome

#### 3.4.1 Effect of surgical technique on GTR

Excluding the 65 biopsy-only cases, GTR was achieved in 67% of conventional craniotomy cases (830/1243), 46% of endoscopic cases (103/223), and 76% of tubular retractor cases (26/34) (Fig. 5, Panel A). The distribution of resection outcomes differed significantly between endoscopic and conventional approaches (46% undergoing GTR in the endoscopic group, 76% in the tubular group versus 67% in the conventional group; 27% undergoing non-GTR resection in the endoscopic group, 24% in the tubular group versus 33% in the conventional group; p < 0.001) but not between tubular and conventional approaches (p = 0.38). No pairwise comparison demonstrated a statistically significant difference in GTR odds after multivariable adjustment: endoscopic vs conventional OR 0.84 (95% CrI 0.26–3.41), tubular vs conventional OR 0.24 (95% CrI 0.02–2.42), and tubular vs endoscopic OR 0.29 (95% CrI 0.03–3.03) (Fig. 5, Panel B).

**Fig. 5:**
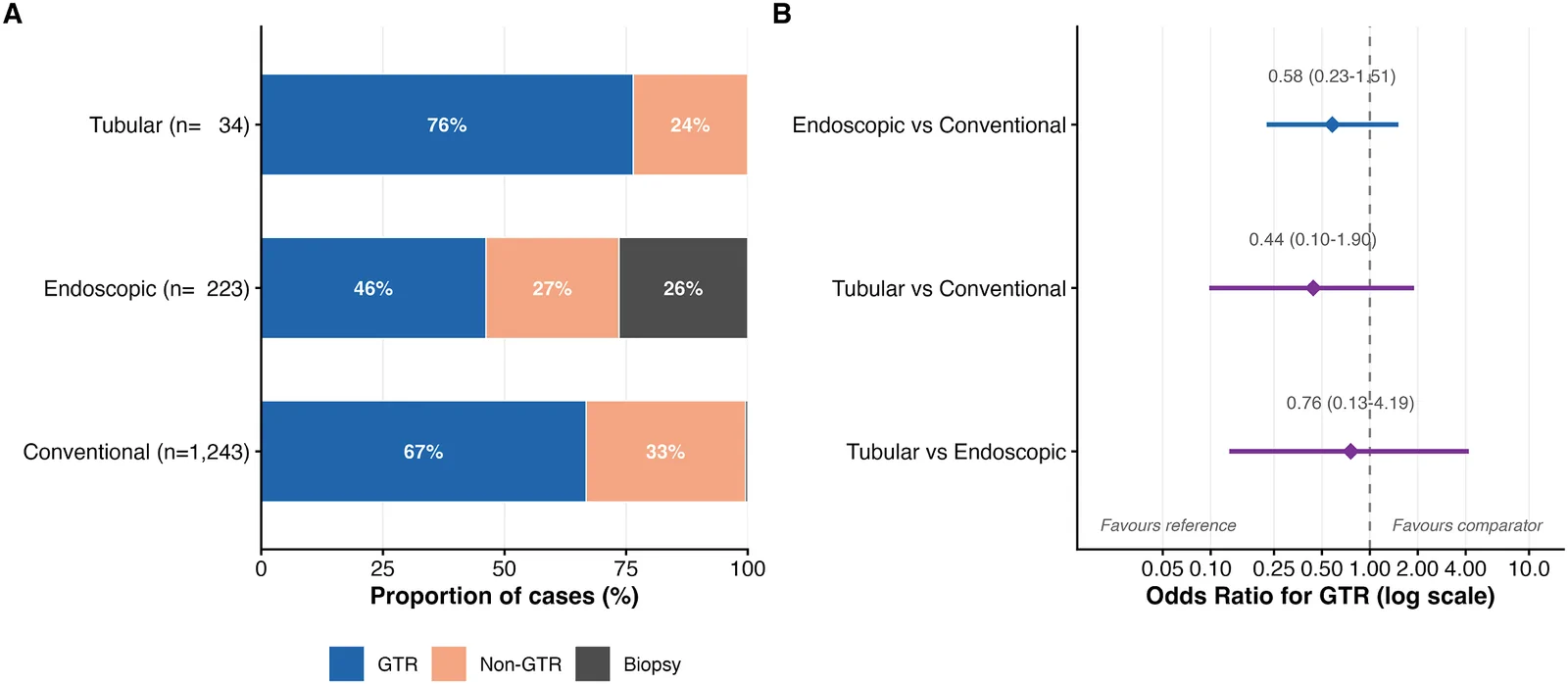
Resection outcomes and adjusted odds ratios for gross total resection (GTR) by surgical approach. Panel A: proportion of GTR, non-GTR, and biopsy cases by surgical technique (resection and biopsy cases; n = 1500). Panel B: adjusted odds ratios (95% CrI) for pairwise comparisons of GTR odds between surgical approaches (resection cases only; multivariable Bayesian hierarchical model; m = 10 imputed datasets). Blue = 95% CrI excludes unity. OR = odds ratio; CrI = credible interval; GTR = gross total resection.

In complete-case univariate models, MIS was associated with lower odds of GTR compared with conventional surgery (OR 0.52, 95% CrI 0.21–1.28); the complete-case multivariable estimate was uninformative due to sparse data (OR 1.13, 95% CrI 0.07–18.60). Imputed univariate analysis produced consistent findings with the complete-case univariate model (OR 0.53, 95% CrI 0.21–1.32). After multivariable adjustment in the imputed dataset, the point estimate moved toward the null (OR 0.43, 95% CrI 0.17–1.08); the credible interval included unity. Between-study heterogeneity in GTR rates was substantial across all models (τ = 2.01–4.1), reflecting considerable variability in case selection, tumour characteristics, and institutional practice across contributing studies (Supplementary Material S4).

#### 3.4.2 Adjusted association of surgical approach with GTR across clinical scenarios

A pre-specified multivariable Bayesian hierarchical logistic regression was performed in oncological intraventricular tumour resection cases (n = 988) to examine the association of surgical approach with GTR across clinically relevant tumour profiles, adjusting for age, tumour diameter, multiplicity, grade, ventricular location, cystic morphology, pre-operative hydrocephalus, and histological category (Supplementary Material S5). Benign and cystic lesions (n = 446), of which 96% were colloid cysts, were excluded due to a GTR rate of 84% producing near-complete separation. Between-study heterogeneity in baseline GTR rate was substantial (τ = 2.01, 95% CrI 1.52–2.62). For scenario-based predictions, small tumours were defined as ≤20 mm diameter, medium as 30 mm, and large as ≥45 mm, corresponding approximately to the 25th percentile, median, and 75th percentile of the observed size distribution, respectively.

Open surgery was associated with higher odds of GTR compared with MIS (OR 0.38, 95% CrI 0.13–1.13; Pd = 96%), and this finding was consistent following propensity score adjustment (OR 0.42, 95% CrI 0.15–1.16; Pd = 95%). Very few contributing studies utilised both approaches within the same series (between-study heterogeneity in treatment effect: τ = 0.72, 95% CrI 0.03–3.29). The E-value for the point estimate was 2.61.

Scenario-based predicted probabilities across eleven pre-specified clinical profiles demonstrated variation in GTR rates by tumour grade, size, location, and multiplicity under both approaches (Supplementary Material S5). The highest predicted GTR rates were observed for Choroid/Embryonal tumours in the lateral ventricle (Open 90%, 95% CrI 12–97%; MIS 76%, 4–95%) and the lowest for large high-grade fourth ventricular tumours with hydrocephalus (Open 49%, 17–85%; MIS 27%, 6–77%).

### 3.5 Secondary outcomes

#### 3.5.1 Independent predictors for GTR

The same multivariable model was used to identify independent clinical predictors of GTR irrespective of surgical approach. Choroid or embryonal histology was the strongest positive predictor compared with glioma (OR 3.38, 95% CrI 0.47–9.27; Pd = 86%). Factors independently associated with lower odds of GTR were cystic tumour morphology (OR 0.40, 95% CrI 0.13–1.12; Pd = 96%), fourth ventricular location compared with lateral ventricle (OR 0.51, 95% CrI 0.23–1.12; Pd = 95%), and tumour multiplicity (OR 0.59, 95% CrI 0.28–1.30; Pd = 91%). Third ventricular location, tumour grade, age, pre-operative hydrocephalus, and tumour diameter were not independently associated with GTR.

#### 3.5.2 Complications

##### 3.5.2.1 Any complications

Complication data were available for 567 of 1500 patients (37.8%) across 225 studies. Among those, any complication occurred in 277 cases (48.9%). Within resection cases, complication rates were 51.0% (239/469) for conventional craniotomy and 49.1% (28/57) for endoscopic approaches; the tubular retractor group showed a lower rate (6/20, 30.0%), though the small sample precludes meaningful comparison. Intraoperative complications were reported exclusively in conventional resection cases. Immediate postoperative complications were the most frequently recorded complication type (213/483, 44.1%), with comparable rates for conventional (46.1%) and endoscopic (50.0%) resection approaches and a lower rate in tubular resection cases (20.0%). Delayed postoperative complications occurred in 80 of 184 cases with recorded data (43.5%) and did not differ significantly across groups (p = 0.151). The distribution of any complication and immediate postoperative complications differed significantly across surgical goal-technique groups (p = 0.004). Given that 62.2% of patients had no recorded complication data and that tumour grade was missing in 77% of patients with available complication data, complication analyses are presented as observed descriptive statistics only; adjusted regression was not performed.

##### 3.5.2.2 Permanent CSF diversion

Permanent CSF diversion data were available for 424 of 1500 patients (28.3%), with diversion performed in 277 cases (65.3%). Biopsy cases required diversion in 97.9% (47/48) compared with 61.2% of resection cases (230/376; p < 0.001). Within resection cases, diversion rates differed significantly by surgical technique (p < 0.001): conventional craniotomy 57.1% (185/324), endoscopic 85.7% (42/49), and tubular 100.0% (3/3). No technique differences were observed within biopsy cases (p = 1.000).

Multivariable mixed-effects logistic regression was performed in resection cases only (n = 376; 230 events). Pre-operative hydrocephalus was the only independent predictor of permanent CSF diversion (OR 8.92, 95% CrI 2.04–38.93). Non-GTR showed a directionally consistent association with increased diversion requirement compared with GTR (OR 6.78, 95% CrI 0.86–53.41); the credible interval crossed unity. Raised ICP at presentation was not independently associated with CSF diversion (OR 1.52, 95% CrI 0.41–5.68). Surgical approach was not independently associated with CSF diversion (OR 2.42, 95% CrI 0.07–85.19). Sex was excluded due to complete separation; tumour grade was excluded due to 83% pre-imputation missingness.

### 3.6 Sensitivity analysis

#### 3.6.1 Modern era (2001-present)

In a sensitivity analysis restricted to studies with observed publication year from 2001 onwards (n = 736 resection cases; 1045 patients had missing year data and were excluded), open surgery was associated with lower odds of GTR compared with MIS, with the credible interval excluding unity (OR 0.35, 95% CrI 0.13–0.95; P(Open > MIS) = 98%). The non-random nature of missing year data may introduce selection bias into this subgroup.

#### 3.6.2 Larger studies only (n > 5)

Restricting analysis to studies contributing more than five patients (68/397 studies; n = 664 oncological resection cases), the directional finding was preserved with the credible interval crossing unity (OR 0.56, 95% CrI 0.15–2.07; P(Open > MIS) = 81%).

### 3.7 Specific scenarios – colloid cyst resection

Among benign and cystic lesions of the third ventricle (n = 247), GTR was achieved in 82% of cases treated with open surgery and 77% with MIS. In the adjusted model accounting for tumour size, age, pre-operative hydrocephalus, and study clustering, there was no credible difference in GTR between approaches (OR 0.73, 95% CrI 0.17–3.03; P(Open > MIS) = 67%; P(within ROPE) = 11%).

## 4 Discussion

We present a large systematic review and meta-analysis on the surgical management of superior intraventricular tumours, with some consistent patterns emerging from our study. In the univariate analysis, the EoR was significantly greater in the conventional surgery group. However, the surgical approach was not significant for the rates of GTR achieved in the multivariable analysis. Permanent CSF diversion was significantly higher in patients undergoing tumour resection with MIS compared with conventional surgery in the univariate analysis. When considering adjustment for patient and tumour factors, the type of surgery performed was not predictive of the need for CSF diversion. In the multivariable analysis, pre-operative hydrocephalus was the only factor that significantly predicted the need for CSF diversion.

### 4.1 Patient and tumour characteristics and type of surgery performed

Patient and tumour characteristics differed between surgical groups, with younger patients being more commonly represented in the conventional craniotomy group compared with MIS (mean age 22 years [10-43] versus 30 years [17-44] for endoscopic and 49 years [32-40] for tubular surgery, p < 0.001). This observation may be due to the slower adoption of endoscopic surgery for larger, more aggressive tumours, which are more common in the younger population (Cinalli et al., 2023; Ruiz-Garcia et al., 2020). Tumours in the conventional group had a larger maximal tumour diameter (31 mm [20–50]) compared to those in the non-conventional (MIS) group (endoscopic 16 mm [13–60] and tubular 12 mm (Desai et al., 2002; Eliyas et al., 2016; Souweidane, 2005; Schroeder, 2013), p < 0.001), suggesting that larger tumours are more likely to undergo conventional craniotomy (Barber et al., 2013). Previous studies have reported different size thresholds for complete resection, suggesting that lesions with diameters under 20 mm are suitable for GTR with endoscopic surgery, while lesions larger than 20 mm should be considered for management with conventional craniotomy, to minimise operative time and reduce associated complications (Souweidane, 2005). However, this threshold is not absolute and extent of resection achieved can be higher in patients with other tumour characteristics, such as non-invasive, lower grade tumours (Chen et al., 2019) and tumours with a cystic component. Indeed, another observed difference between the conventional and MIS group was the higher incidence of tumours with a cystic component in the MIS group (94% for both endoscopic and tubular surgery vs 73%), indicating that cystic tumours were significantly over-represented in the MIS group. However, in absolute terms, most cystic tumours underwent conventional craniotomy (n = 187/298 versus n = 109/298, p < 0.001). Previous literature has suggested that cystic tumours have significantly higher rates of complete resection as they are more amenable to debulking via MIS (Barber et al., 2013).

### 4.2 Surgical approach and achievement of GTR

Most patients in the included studies had conventional craniotomy (n = 1243; 83%), likely reflecting historical practice and the more recent adoption of MIS. Conventional craniotomy had the highest GTR rates (n = 830, 67%; p < 0.001 in the univariate analysis) among surgical approaches. However, there was substantial between-study heterogeneity and our meta-analysis, adjusting for age, tumour maximal diameter, multiplicity, grade, ventricular location, cystic morphology, pre-operative hydrocephalus, and histological category, revealed that there was no statistically significant difference in achieving GTR following conventional and MIS approaches. This was replicated in a sensitivity analysis limited to more populated studies (reporting details in over five cases), showing there was no preference between MIS or conventional craniotomy. When a sensitivity analysis was conducted for studies published after 2001, conventional surgery had lower odds of achieving GTR than MIS, suggesting that in more contemporary series, MIS may confer a significant advantage in achieving total resection. However, as the year of population was missing for a large proportion of patients and some of the included publications reported on cohorts that extended before 2001, this subgroup analysis is susceptible to selection bias so findings should be interpreted cautiously. Overall, these findings suggest that case selection, tumour biology and surgeon preference may play a more important role in the choice of approach and extent of resection achieved than the characteristics of the surgical approach. Nevertheless, our multivariable model of predictors for GTR revealed that while choroidal/embryonal lesions and low-grade tumours are more likely to be completely resected, this did not reach statistical significance. Given that no predictor of GTR reached statistical significance, the choice of surgical approach depends on individual case presentation (Darkwah et al., 2017).

### 4.3 Surgical approach and complication rates

Surgical complications were reported in 37.8% of patients (n = 567/1500), and among those, complications at any point were observed in 48.9% (n = 277), a relatively high rate compared with the overall complication incidence for neurosurgical procedures (∼14%) (Rolston et al., 2014). Complication data was only reported in 567 patients across the included studies, so missing data may lead to poor estimation of the true complication rate. Previous systematic reviews focusing on outcomes after endoscopic intraventricular surgery or in paediatric intraventricular gliomas have reported lower rates (20.8%-28%) (Barber et al., 2013; Watanabe et al., 2024). This increased risk of complications compared with other neurosurgical procedures is largely attributed to the challenges of intraventricular tumour surgery, which involves deep-seated targets, neighbouring critical structures and narrow surgical corridors (Verducci et al., 2024). In this review, complication rates for resection surgery were 51% (n = 239) in the conventional cohort compared to 49.1% (n = 28) and 30% (n = 6) in the minimally invasive groups. Given that a large proportion of patients did not have available complication data, no formal regression analyses were conducted to demonstrate whether conventional or MIS is significantly associated with a higher risk profile. Previous studies have suggested that MIS is not associated with a statistically significant difference in complication risk compared to conventional surgery (Verducci et al., 2024; Farahmand et al., 2023). Furthermore, the influence of surgeon sub-specialisation and case volume on complications was not explored. These limitations highlight the need for further prospective studies to determine whether MIS and conventional approaches have comparable complication profiles.

### 4.4 Surgical approach and CSF diversion

MIS had a higher incidence of permanent CSF diversion; however, this association did not persist in the biopsy-only cases group and following adjustment for risk factors for post-operative hydrocephalus. This initial observation is likely to be driven by baseline selection bias, as MIS was performed in patients with pre-operative hydrocephalus (35% in conventional group versus 39% in endoscopic surgery and 57% in tubular surgery) or a more challenging anatomy. The multivariable regression analysis demonstrated that the only factor remaining significant for the requirement of CSF diversion was pre-operative hydrocephalus. In such cases, prophylactic EVD placement or concomitant strategies such as ventriculostomy could be considered (Chen et al., 2020).

## 5 Limitations

This review comes with limitations. Data was collected over a long period, during which surgical practice and technology evolved; as a result, the pooled data may not accurately reflect current neurosurgical practice. Furthermore, despite the use of imputation, missing data remained a challenge which could influence the robustness of effect estimates. Many of the included studies had small population sizes, often fewer than 5 patients per series, therefore introducing publication bias. This meta-analysis was not designed to detect changes in the quality of life, cognitive performance or patient-reported outcomes, which are increasingly recognized as important outcomes in the neuro-oncology field. (Dirven et al., 2018; Sanikommu et al., 2026)^,^ (Soleman and Guzman, 2020) Additionally, it was underpowered to detect changes in the white matter microstructure and connectivity resulting from different surgical approaches, which is a potential advantage of MIS techniques (Eliyas et al., 2016). Finally, the effects of hemispheric laterality and dominance, along with the choice of surgical lobar approach (i.e. frontal versus parietal) on primary and secondary outcomes, could also not be reliably assessed due to scarce reporting of this data in the reports included in the meta-analysis (Yaşargil and Abdulrauf, 2008).

## 6 Conclusion

This systematic review and meta-analysis show that conventional and MIS approaches for intraventricular tumours achieve comparable rates of GTR, with no approach demonstrating clear superiority over the other. Specifically, while there were significantly more patients undergoing GTR in the conventional surgery group in the univariate analysis, this association was not sustained after multivariable adjustment. Overall complication rates of intraventricular tumour surgery are high and sparsely reported in the literature. An increased rate of permanent CSF diversion was observed on univariate analysis; however this was not supported by multivariable analysis.

## Author contributions

The authors of the study made the following contributions for its publication:

ABM: conceptualization, formal analysis, investigation, writing- original draft, writing-review& editing, visualization, supervision.

AG: conceptualization, methodology, investigation, writing- original draft, writing-review& editing, visualization.

SR: methodology, formal analysis, writing- original draft, writing-review& editing, visualization.

JP: investigation, writing- original draft.

FF: methodology, formal analysis, writing-review& editing.

PYL: investigation, formal analysis.

AV: investigation.

NS: investigation.

SZ: investigation.

YAC: conceptualization, investigation, writing-review& editing, visualization.

KA: conceptualization, writing-review& editing.

FV: conceptualization, writing-review& editing.

RB: conceptualization, writing-review& editing.

BZ: conceptualization, writing-review& editing.

CB: conceptualization, writing-review& editing.

JPL: conceptualization, methodology, writing- original draft, writing-review& editing, vizualisation, supervision.

## Data availability

Data will be made available upon reasonable request.

## Ethics approval

Ethical approval was not required for this study as it is a systematic review.

## Funding

This research did not receive any specific grant from funding agencies in the public, commercial, or not-for-profit sectors.

## Declaration of competing interests

The authors declare that they have no known competing financial interests or personal relationships that could have appeared to influence the work reported in this paper.
