# Supplementary material for: Intraventricular tumours: An individual patient data meta-analysis of conventional craniotomy versus minimally invasive techniques
Source: Brain Spine. 2026 Oct 6;6:107467. doi: 10.1016/j.bas.2026.107467 (PMC13720992; doi:10.1016/j.bas.2026.107467)
Supplement: Supplementary file 2 [file mmc2.docx]

**Supplementary Material S1. Search strategies per database**

| **Embase (n=12,397)** | #1 ‘intraventricular’ OR ‘ventricular’  #2 ‘tumour’ OR ‘tumor’  #3 ‘cardiac’  #4 #1 and #2  #5 #4 NOT #3  From inception-November 2024 |
| --- | --- |
| **Web of Science (n=5,621)** | All Databases search  TS=(((intraventricular OR ventricular) AND (tumour OR tumor OR neoplasm)) NOT (cardiac or heart))  From inception-November 2024 |
| **Pubmed (n=9,662)** | (intraventricular OR ventricular) AND (tumour OR tumor) NOT (cardiac)  From inception-November 2024 |
| **Scopus (n=8,308)** | ( TITLE-ABS-KEY ( ( intraventricular OR ventricular ) ) AND TITLE-ABS-KEY ( ( tumour OR tumor OR neoplasm ) ) AND NOT TITLE-ABS-KEY ( ( cardiac OR heart ) ) )  From inception-November 2024 |
| **Cochrane (n=792)** | #1 (intraventricular OR ventricular):ti,ab,kw  #2 (tumour OR tumor OR neoplasm):ti,ab,kw  #1 and #2  From inception-November 2024 |

**Supplementary Material S2. Sensitivity Analyses – MIS vs Open Surgery for GTR**

| Analysis | N | Adjusted OR (95% CrI) | P(Open > MIS) |
| --- | --- | --- | --- |
| Primary model (oncological, full cohort) | 1435 | 0.48 (0.19-1.16) | 95% |
| Modern era (2001+, observed year only) | 1164 | 0.51 (0.20-1.29) | 92% |
| Large studies only (n>5) | 959 | 0.73 (0.21-2.49) | 70% |
| Overlap-adjusted (one study per author) | 1324 | 0.58 (0.23-1.47) | 88% |
| Comparative studies only (within-study) | 39 | 0.96 (0.19-3.95) | 52% |

**Supplementary Material S3. Propensity score overlap between surgical approaches**

*Propensity score overlap between surgical approaches. Kernel density distributions of the propensity score (predicted probability of receiving MIS) for patients undergoing open (blue) and MIS (red) surgery. Propensity scores were estimated using a logistic regression model including tumour diameter, age, tumour grade, multiplicity, ventricular location, pre-operative hydrocephalus, cystic morphology, and histological category as predictors. Substantial overlap between distributions is required for valid propensity score adjustment. The observed separation — with open surgery concentrated at low propensity scores — confirms preferential selection of MIS for cases judged anatomically accessible, indicating significant confounding by surgical indication*

**Supplementary Material S4. Baseline characteristics stratified by surgical approach (imputed data)**

| Characteristic | N | **Overall**  N=1,500 | **Conventional**  N=1,243 | **Endoscopic**  N=223 | **Tubular**  N=34 | p (Conv vs MIS) | p (Conv vs Endoscopic) | p (Conv vs Tubular) |
| --- | --- | --- | --- | --- | --- | --- | --- | --- |
| Extent of resection: Biopsy | 1500 | 65 (4.3%) | 6 (0.5%) | 59 (26.5%) | 0 (0.0%) | **<0.001** | **<0.001** | 0.382 |
| Extent of resection: GTR | 1500 | 959 (63.9%) | 830 (66.8%) | 103 (46.2%) | 26 (76.5%) | **<0.001** | **<0.001** | 0.382 |
| Extent of resection: Non-GTR | 1500 | 476 (31.7%) | 407 (32.7%) | 61 (27.4%) | 8 (23.5%) | **<0.001** | **<0.001** | 0.382 |
| Age | 1500 | 25 (12, 44) | 23 (10, 43) | 30 (17, 44) | 48 (32, 60) | **<0.001** | **<0.001** | **<0.001** |
| Baseline KPS | 174 | 90 (70, 100) | 90 (70, 100) | NA (NA, NA) | 80 (80, 90) | 0.794 | - | 0.794 |
| Tumour size (mm) | 1500 | 30 (16, 50) | 30 (16, 50) | 40 (16, 62) | 12 (9, 20) | 0.143 | **<0.001** | **<0.001** |
| Length of stay | 116 | 6 (4, 10) | 6 (5, 10) | 8 (5, 8) | 4 (2, 5) | **0.008** | 0.497 | **<0.001** |
| PFS | 51 | 2 (1, 7) | 2 (1, 6) | 5 (2, 7) | 12 (12, 12) | 0.356 | 1.000 | - |
| Follow-up (years) | 805 | 2 (1, 4) | 2 (1, 4) | 1 (1, 2) | 0 (0, 0) | **<0.001** | **<0.001** | **<0.001** |
| Tumour grade: High Grade | 1500 | 298 (19.9%) | 267 (21.5%) | 24 (10.8%) | 7 (20.6%) | **<0.001** | **<0.001** | 1.000 |
| Tumour grade: Low Grade | 1500 | 1202 (80.1%) | 976 (78.5%) | 199 (89.2%) | 27 (79.4%) | **<0.001** | **<0.001** | 1.000 |
| Permanent CSF diversion | 424 | 277 (65.3%) | 186 (57.2%) | 88 (91.7%) | 3 (100.0%) | **<0.001** | **<0.001** | 0.265 |
| Male | 1500 | 723 (48.2%) | 589 (47.4%) | 117 (52.5%) | 17 (50.0%) | 0.171 | 0.167 | 0.862 |
| Cystic component | 1500 | 1054 (70.3%) | 837 (67.3%) | 190 (85.2%) | 27 (79.4%) | **<0.001** | **<0.001** | 0.192 |
| Pre-op hydrocephalus | 1500 | 533 (35.5%) | 420 (33.8%) | 92 (41.3%) | 21 (61.8%) | **0.003** | **0.033** | **0.001** |
| Raised ICP symptoms | 1500 | 728 (48.5%) | 598 (48.1%) | 128 (57.4%) | 2 (5.9%) | 0.493 | **0.011** | **<0.001** |
| Median (Q1, Q3) for continuous variables; n (%) for categorical variables. Percentages represent proportion within each surgical approach. Fisher's exact test for categorical variables; Wilcoxon rank-sum for continuous variables. | | | | | | | | |

**Supplementary Material S5. Predicted Gross Total Resection Rates and Absolute Risk Difference for Open versus MIS across 11 pre-specified clinical scenarios**

*Predicted P(GTR) (Panel A) and absolute risk difference — Open minus MIS (Panel B) — across eleven pre-specified clinical scenarios (Bayesian hierarchical model; population-average predictions). Green shading in Panel B denotes 0–10% (light), 10–20% (medium), and 20–30% (dark) absolute difference bands. Age held at population mean; remaining covariates at reference values. τ (MIS treatment effect) = 1.40 (95% CrI 0.06–4.27).*

**Supplementary Material S6. Posterior predictive check for the primary GTR predictor model.**

*The observed distribution of gross total resection (thick dark line) is overlaid on 100 outcome distributions simulated from the posterior of the fitted model (thin light lines). Substantial overlap indicates that the fitted model reproduces the observed outcome distribution and shows no evidence of systematic misfit.*
